# Multimechanistic Monoclonal Antibodies (MAbs) Targeting Staphylococcus aureus Alpha-Toxin and Clumping Factor A: Activity and Efficacy Comparisons of a MAb Combination and an Engineered Bispecific Antibody Approach

**DOI:** 10.1128/AAC.00629-17

**Published:** 2017-07-25

**Authors:** C. Tkaczyk, S. Kasturirangan, A. Minola, O. Jones-Nelson, V. Gunter, Y. Y. Shi, K. Rosenthal, V. Aleti, E. Semenova, P. Warrener, D. Tabor, C. K. Stover, D. Corti, G. Rainey, B. R. Sellman

**Affiliations:** a Medimmune LLC, Gaithersburg, Maryland, USA; b Humabs, Bellinzona, Switzerland

**Keywords:** Staphylococcus aureus, alpha toxin, clumping factor A, monoclonal antibodies

## Abstract

Secreted alpha-toxin and surface-localized clumping factor A (ClfA) are key virulence determinants in Staphylococcus aureus bloodstream infections. We previously demonstrated that prophylaxis with a multimechanistic monoclonal antibody (MAb) combination against alpha-toxin (MEDI4893*) and ClfA (11H10) provided greater strain coverage and improved efficacy in an S. aureus lethal bacteremia model. Subsequently, 11H10 was found to exhibit reduced affinity and impaired inhibition of fibrinogen binding to ClfA002 expressed by members of a predominant hospital-associated methicillin-resistant S. aureus (MRSA) clone, ST5. Consequently, we identified another anti-ClfA MAb (SAR114) from human tonsillar B cells with >100-fold increased affinity for three prominent ClfA variants, including ClfA002, and potent inhibition of bacterial agglutination by 112 diverse clinical isolates. We next constructed bispecific Abs (BiSAbs) comprised of 11H10 or SAR114 as IgG scaffolds and grafted anti-alpha-toxin (MEDI4893*) single-chain variable fragment to the amino or carboxy terminus of the anti-ClfA heavy chains. Although the BiSAbs exhibited *in vitro* potencies similar to those of the parental MAbs, only 11H10-BiSAb, but not SAR114-BiSAb, showed protective activity in murine infection models comparable to the respective MAb combination. *In vivo* activity with SAR114-BiSAb was observed in infection models with S. aureus lacking ClfA. Our data suggest that high-affinity binding to ClfA sequesters the SAR114-BiSAb to the bacterial surface, thereby reducing both alpha-toxin neutralization and protection *in vivo*. These results indicate that a MAb combination targeting ClfA and alpha-toxin is more promising for future development than the corresponding BiSAb.

## INTRODUCTION

Infections caused by antimicrobial-resistant (AMR) bacterial pathogens are an increasing threat to public health. The ongoing AMR epidemic has been fueled, in part, by empirical broad-spectrum antibiotic therapy (1). This has led to the exploration of pathogen-specific methods, including the use of monoclonal antibodies (MAbs), to prevent or treat serious bacterial infections. Numerous MAbs are currently in development for the prevention or treatment of antibiotic-resistant bacterial infections (2). Such passive immunization strategies provide an immediate and potent IgG response against the target pathogen. Ideally, the MAb, or MAb cocktail, provides multiple mechanisms of action to neutralize key bacterial virulence factors and augment the host innate immune response, thus providing the greatest opportunity for clinical success.

Staphylococcus aureus is a bacterial pathogen that causes a wide array of diseases, including skin and soft-tissue infections, endocarditis, osteomyelitis, pneumonia, and bacteremia (3). Preclinical results indicate MAb-based approaches hold promise for prophylaxis and adjunctive therapy against S. aureus infections (4–8). We previously reported that prophylaxis with a multimechanistic MAb combination targeting alpha-toxin (MEDI4893*) and clumping factor A (ClfA; 11H10) provided enhanced protection and improved strain coverage relative to the individual MAbs in an S. aureus lethal bacteremia model (9). A MAb combination such as this provides multiple mechanisms of action, including toxin neutralization, opsonophagocytic killing, and inhibition of fibrinogen binding and bacterial agglutination. Similarly, a MAb combination targeting Pseudomonas aeruginosa exopolysaccharide Psl and type 3 secretion system component PcrV provided enhanced protection relative to the individual MAbs in a P. aeruginosa acute pneumonia model (10) by mediating opsonophagocytic killing (OPK), blocking cell attachment, and inhibiting the injection of multiple virulence factors into target cells. These two examples provide support for multimechanistic MAb-based antibacterial treatment approaches.

An alternative approach to a MAb combination is to engineer both binding specificities into a single bispecific (BiS) or multispecific IgG molecule (11). The first BiS antibodies (BiSAbs) generated by somatic hybridization of two antibody-secreting cells were produced with poor yield due to random assembly of parental heavy and light chains (12). The discovery of single-chain variable fragments (scFvs) and advances in antibody engineering have opened new avenues for the development of BiS molecules (13, 14). There are now at least 50 different BiSAb formats based on scFv numbers and fusion positions on the IgG scaffold (15). One clear mechanistic advantage of a BiSAb can arise when binding of one specificity facilitates the binding and activity of the second specificity. This has been observed with the BiSAb MEDI3902, which targets the P. aeruginosa cell surface exopolysaccharide Psl and the tip of the type 3 secretion system injectisome, PcrV. In MEDI3902, the anti-Psl scFv was engineered into the hinge region of an anti-PcrV IgG1. Interestingly, this construct provided enhanced protection relative to the anti-PcrV–anti-Psl MAb combination in a P. aeruginosa acute pneumonia model (10). The improved activity was hypothesized to result from MEDI3902 high-avidity, lower-affinity binding to the abundant Psl polysaccharide around the bacterium, effectively increasing the concentration of the higher-affinity anti-PcrV MAb around the cell. Based on these results, we hypothesized that high-affinity binding of a BiSAb comprised of binding specificities for ClfA and alpha-toxin could increase the protective capacity of the MAb combination by localizing the anti-alpha-toxin specificity on the bacterial surface, better enabling the BiSAb to neutralize the toxin upon its secretion.

Here, we generated various BiSAbs containing anti-alpha-toxin and anti-ClfA activities. We determined that the anti-ClfA MAb 11H10 exhibited poor binding affinity for a predominant ClfA sequence type (ClfA002) and consequently generated a new anti-ClfA MAb, SAR114, with increased affinity for the three main ClfA sequence types (16). Anti-ClfA plus anti-alpha-toxin BiS molecules were comprised of 11H10 or SAR114 and an anti-alpha-toxin, MEDI4893*, and their relative potencies were compared *in vitro* and *in vivo*. Although the BiS molecules constructed from 11H10 and SAR114 retained the *in vitro* potency of the parental MAbs, the BiS molecules constructed from the higher-affinity anti-ClfA MAb SAR114 exhibited reduced protective activity *in vivo* relative to the MAb combination in S. aureus pneumonia and bacteremia models. In contrast, the *in vivo* activity of the 11H10 BiSAbs was comparable to that observed with the respective MAb combinations. Interestingly, SAR114-BiS protective activity was evident in mice challenged with an isogenic mutant defective for ClfA expression (SF8300Δ*clfA*), suggesting that SAR114 high-affinity binding to ClfA in the BiS molecule sequesters MEDI4893* on the bacterial surface, thereby preventing alpha-toxin neutralization. These results indicate that a BiSAb approach is not always superior to a MAb combination, and appropriate platform selection requires rigorous preclinical studies in relevant animal models.

## RESULTS

### Characterization of anti-ClfA MAb SAR114.

**FIG 1:**
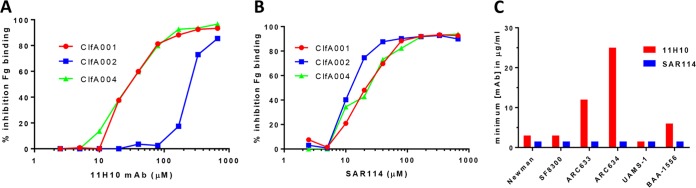
SAR114 MAb broad reactivity against the three main ClfA genotypes *in vitro*. Inhibition of fibrinogen binding to the three main ClfA genotypes was measured in the presence of serially diluted (from 666 μM to 2.55 μM) anti-ClfA MAb 11H10 (A) or SAR114 (B). Data are representative of three independent experiments. (C) Agglutination of S. aureus clinical isolates in the presence of human plasma. The graph shows the minimum concentration of 11H10 (■) and SAR114 (■) required to inhibit bacterial agglutination. Data are representative of two independent experiments. c-IgG, used as a negative control, did not show any inhibition at 200 μg/ml.

**FIG 2:**
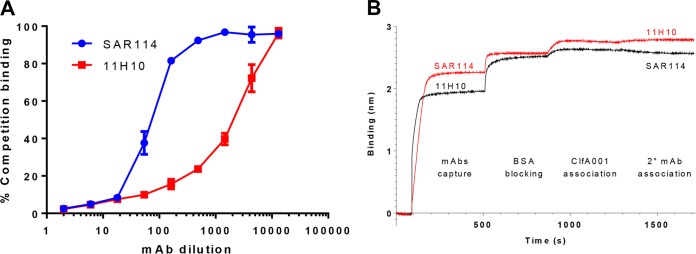
SAR114 and 11H10 share an overlapping epitope on ClfA001 genotype. SAR114 and 11H10 compete for binding to the ClfA001 genotype. (A) ELISA competition binding. Serial dilution (1:2 or 1:600) of SAR114 (■) or 11H10 (■) was added to ClfA-coated plates, followed by addition of biotinylated 11H10 (1:600). Percent competition was calculated as 100 × (OD_MAb + 11H10biot_/OD_11H10biot_). Data represent the mean values ± standard deviations (SD). (B) Octet binding. SAR114 (•) or 11H10 (■), diluted at 5 μg/ml, was captured on an APS biosensor and blocked with BSA, followed by addition of ClfA001 (2.5 μg/ml), and then a second MAb diluted at 5 μg/ml was added. Real-time measurement of the binding to the biosensors (in nanometers) was registered.

We previously reported on the enhanced protective capacity and isolate coverage afforded by prophylaxis with an anti-S. aureus alpha-toxin MAb (MEDI4893*) in combination with an anti-ClfA MAb (11H10) relative to the individual MAbs in an S. aureus lethal bacteremia model (9). Although 11H10 is a potent anti-ClfA MAb, we found it exhibited a >1,000-fold reduced affinity (*K*_on_; below the limit of detection; ND in Table 1) and an ∼40-fold increase in the 50% inhibitory concentration (IC_50_) for ClfA002 in the fibrinogen binding inhibition assay relative to ClfA001 and ClfA004 (Fig. 1A and Table 1). ClfA002 is expressed by a prominent S. aureus hospital-acquired methicillin-resistant S. aureus (HA-MRSA; USA100 or sequence type 5 [ST5]) strain (17, 18). To increase potential clinical isolate coverage, we screened human tonsillar B cells to search for more broadly reactive anti-ClfA MAbs. From this effort, we identified a MAb (SAR114) with high affinity for ClfA001, ClfA002, and ClfA004 (apparent dissociation constant [*K_D_*] of 1.15 to 44.7 pM) (Table 1) and potent inhibition of fibrinogen binding by these 3 prominent ClfA genotypes (IC_50_ of ∼20 μM) (Fig. 1B). SAR114 also exhibited opsonophagocytic killing (OPK) activity against several S. aureus clinical isolates (see Fig. S1 in the supplemental material) and improved inhibition of bacterial agglutination in human plasma compared to 11H10 (Fig. 1C; Fig. S2). 11H10 and SAR114 were found to compete for binding to ClfA001 by enzyme-linked immunosorbent assay (ELISA) and in an Octet-based competition assay (Fig. 2A and B). These results indicate the MAbs bind an overlapping epitope on ClfA001, suggesting their potency difference against ClfA002 results from different binding affinities.

**TABLE 1:** Anti-ClfA MAbs: correlation between affinity and *in vitro* activity

Antibody and ClfA variant	Affinity^*a*^			Fibrinogen binding^*b*^ (IC_50_ [μM])
	*K*_on_ (M^−1^ s^−1^)	*K*_off_ (s^−1^)	*K_d_*	
SAR114				
ClfA001	2.41E+06	6.01E−06	2.493 pM	16.09
ClfA002	2.13E+06	9.53E−05	44.77 pM	6.004
ClfA004	5.62E+06	6.46E−06	1.15 pM	13.61
11H10				
ClfA001	1.09E+06	6.80E−03	6.22 nM	24.59
ClfA002			ND	997.5
ClfA004	8.45E+05	6.39E−03	7.55 nM	23.94

a Affinity constants (*K_d_*) of SAR114 and 11H10 to the main ClfA variants were determined as the ratio between dissociation (*K*_off_) and association (*K*_on_) rate constants, as measured by Biacore. The affinity of 11H10 to ClfA002 could not be determined (ND).

b *In vitro* activity of both anti-ClfA MAbs was measured in the fibrinogen binding inhibition assay for the main three ClfA variants and is expressed as IC_50_.

### SAR114-MEDI4893* MAb combination provides broad strain coverage in lethal bacteremia.

SAR114 protective activity next was evaluated in a murine lethal bacteremia model, alone and in combination with MEDI4893*. SAR114 (15 mg/kg) prophylaxis resulted in increased survival compared to an isotype control IgG (c-IgG) following challenge with S. aureus isolates representing ST8, ST5, or ST30, confirmed to encode ClfA genotypes ClfA001, ClfA002, and ClfA004, respectively (Table 2; Fig. S3). Similar to 11H10-MEDI4893*, prophylaxis with the SAR114-MEDI4893* combination (7.5 mg/kg each) significantly increased survival relative to c-IgG following challenge with all strains tested and provided a benefit over the individual MAbs against some strains (Table 2) (9). These results indicate SAR114 is functional *in vivo* and confirms the need for a MAb combination to provide broader strain coverage.

**TABLE 2:** SAR114-MEDI4893* MAb combination strain coverage protection in lethal bacteremia

Strain	ST	Protection against lethal bacteremia by^*a*^:		
		SAR114 (15 mg/kg)	MEDI4893* (15 mg/kg)	Combination MAb (7.5 mg/kg each)
NRS123	1	+	−	+
ARC2784	188	+	+	+
ARC635	5	+	+	+
NRS387	5	+	+	+
SF8300	8	+	+	+
NRS384	8	−	−	+
3049057	8	−	−	+
3049088	30	+	−	+
3049114	30	+	−	+

a A plus sign indicates significantly different from c-IgG (15 mg/kg) by log rank (Mantel-Cox) test.

### Anti-alpha-toxin–anti-ClfA-bispecific MAb *in vitro* efficacy.

**FIG 3:**
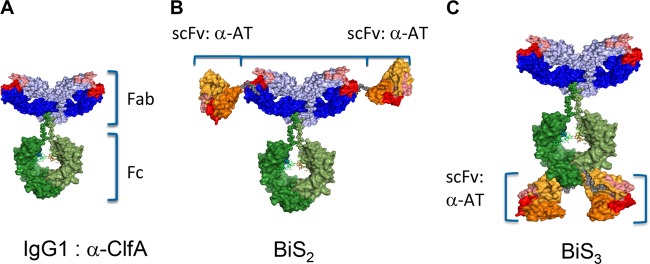
ClfA-alpha-toxin bispecific antibodies compared to IgG1. (A) Bispecific constructs using anti-ClfA MAb as a scaffold. scFv of anti-alpha-toxin MAb MEDI4893* were linked via a 10-amino-acid linker (GGGGx2) to the ClfA MAb heavy-chain N terminus (B) or heavy-chain C terminus (C).

**FIG 4:**
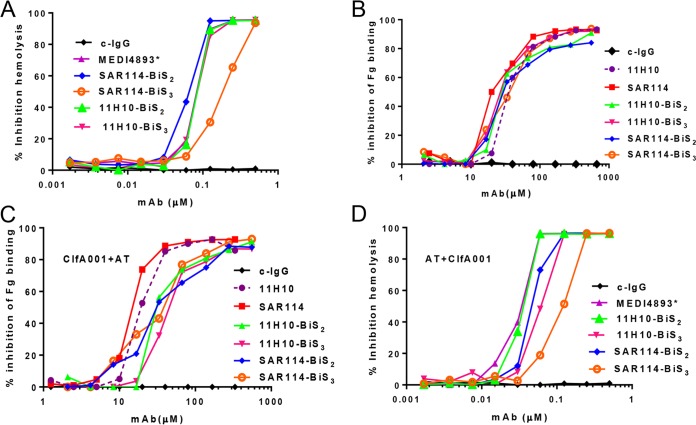
*In vitro* characterization of anti-ClfA SAR114 or 11H10/MEDI4893* BiSAbs. (A) BiS_2_ and BiS_3_ activities compared to that of MEDI4893* in an alpha-toxin-mediated rabbit RBC hemolytic assay. Serial dilutions of BiS_2_, BiS_3_, and MEDI4893* were incubated with purified alpha-toxin and rabbit RBC. Hemolysis was measured by the amount of hemoglobin released in the supernatant. Percent hemolysis inhibition was calculated as 100 × [100 − (OD_alpha-toxin+MAb_/OD_alpha-toxin alone_)]. Data are representative of three independent experiments. (B) Anti-ClfA MAb SAR114 or 11H10 in an immobilized fibrinogen binding assay. ClfA binding to fibrinogen was measured in the presence of serial dilutions (200 to 0.5 μM) of BiS2, BiS3, and SAR114 or a nonspecific c-IgG MAb. Data represent the mean values and standard deviations from three separate experiments. Percent inhibition binding was calculated as 100 × [100 − (OD_ClfA+MAb_/OD_ClfA alone_)].

Passive immunization with an anti-ClfA–anti-alpha-toxin MAb combination provided a benefit for strain coverage in lethal bacteremia and retained the anti-alpha-toxin protective capacity in murine dermonecrosis and pneumonia models (9). To determine if a BiSAb comprised of the anti-ClfA and anti-alpha-toxin MAbs provided benefit over a MAb combination, we engineered BiSAbs comprised of 11H10 or SAR114 as the IgG backbones, to which the MEDI4893* scFv was appended to the amino (BiS_2_) or carboxy (BiS_3_) terminus of the heavy chain (Fig. 3) (19). The BiS_2_ and BiS_3_ formats were selected because the scFv is located in disparate locations on the IgG, and the only way to determine if one format has an advantage over another is to test them empirically for the antibody specificities of interest. To understand if the BiSAbs retained the functional activities of the individual MAbs, we measured their potency in inhibiting alpha-toxin-dependent rabbit red blood cell (RBC) lysis and inhibition of fibrinogen binding to ClfA001, ClfA002, and ClfA004. The 11H10-BiS_2_ and -BiS_3_ molecules and SAR114-BiS_2_ exhibited IC_50_s similar to that of MEDI4893* in an alpha-toxin hemolytic assay, whereas SAR114-BiS_3_ exhibited reduced alpha-toxin neutralization activity (Fig. 4A; Table S2). Both 11H10 BiSAbs and the SAR114 BiS_3_Ab exhibited IC_50_s similar to the respective parental anti-ClfA IgG in the fibrinogen binding inhibition assay, whereas the SAR114 Bis_2_Ab lost some activity against ClfA002 but was still superior to 11H10 (Fig. 4B; Fig. S4 and Table S2). The BiSAbs also mediated OPK similar to that of the parental anti-ClfA IgG (Fig. S5). Importantly, saturation of the anti-alpha-toxin scFv in the presence of a 10 M excess of alpha-toxin did not interfere with anti-ClfA activity in the fibrinogen binding assay (Fig. 4C). Similarly, saturation of ClfA binding with a 10 M ClfA excess did not decrease alpha-toxin neutralizing activity of the BiSAbs in the hemolytic assay (Fig. 4D; Table S2). Taken together, these results indicate the anti-alpha-toxin–anti-ClfA BiS molecules retain *in vitro* functional activity that in most cases was similar to that of the parental IgG, and this activity was not diminished in the presence of a 10-fold molar excess of the other antigen recognized by the BiSAb.

### Anti-alpha-toxin–anti-ClfA BiSAb molecule efficacy in lethal bacteremia.

**FIG 5:**
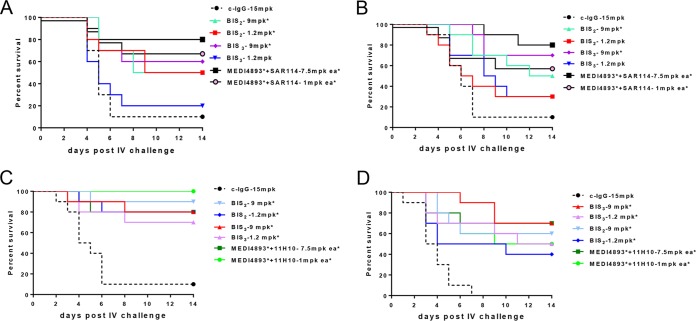
Efficacy for anti-ClfA MAb/MEDI4893* BiSAbs in i.v. bacteremia. BALB/c mice (*n* = 10) were passively immunized i.p. with SAR114/MEDI4893* BiS_2_, BiS_3_, or an SAR114-MEDI4893* combination at the indicated concentrations and were i.v. infected 24 h later with the LD_90_ of SF8300 (6e7 CFU) (A) and 3049057 (5e7 CFU) (B). Protective efficacy for 11H10-MEDI4893* BiS_2_, BiS_3_, or 11H10-MEDI4893* MAbs was evaluated against SF8300 (C) or 3049057 (D) challenge. Survival was monitored for 2 weeks. Results were analyzed with a log rank (Mantel-Cox) test. Statistical analysis of results compared to those for c-IgG were considered statistically different at a *P* value of <0.05 and are indicated with an asterisk. Data are representative of three independent experiments.

The anti-ClfA–anti-alpha-toxin BiSAb protective activities next were compared to those of the MAb combinations in an S. aureus lethal bacteremia model. Mice were passively immunized with the MAb combinations (7.5 mg/kg or 1 mg/kg each) or equimolar doses of the BiSAbs (9 or 1.2 mg/kg, respectively) 24 h prior to intravenous (i.v.) infection with S. aureus SF8300, and survival was monitored for 14 days. Both SAR114-BiSAbs at 9 mg/kg exhibited reduced but not significantly different (*P* = 0.234 for BiS_2_ and *P* = 0.412 for BiS_3_) protection compared to the MAb combination at 7.5 mg/kg each (Fig. 5A). The MAb combination at 1 mg/kg (*P* = 0.0051 versus c-IgG) and SAR114-BiS_2_ at 1.2 mg/kg (*P* = 0.0336 versus c-IgG) significantly increased survival relative to c-IgG. Consistent with the observed loss of alpha-toxin neutralization activity *in vitro* (Fig. 4A), SAR114-BiS_3_ did not significantly increase survival when administered at 1.2 mg/kg (*P* = 0.657) (Fig. 5A). When tested against S. aureus strain 3049057 (MRSA, ST8), a strain where neither MAb alone is sufficient for significant protection (Fig. S3), the SAR114-BiS molecules at 1.2 mg/kg did not significantly increase survival relative to c-IgG (*P* = 0.4310), whereas an equimolar concentration (1 mg/kg) of the MAb combination did (*P* = 0.0348 versus c-IgG) (Fig. 5B). This suggested a defect in the SAR114-BiS_2_Ab *in vivo*. Interestingly, passive immunization with the 11H10-BiSAbs resulted in protection similar to that of the MAb combination at both doses (9 mg/kg and 1.2 mg/kg) and provided a significant increase in survival relative to c-IgG against both ClfA001-expressing strains, SF8300 and 3049057 (Fig. 5C and D). These results indicate that the anti-ClfA–anti-alpha-toxin BiSAbs do not provide benefit over the MAb combination, as was previously observed for the BiS_4_αPA (MEDI3902) against P. aeruginosa; rather, conversely, the SAR114–MEDI4893* BiS exhibited a loss in protection at lower doses against a strain where the individual MAbs are not sufficient to provide protection.

### Binding to ClfA impairs BiSAb efficacy in lethal pneumonia.

**FIG 6:**
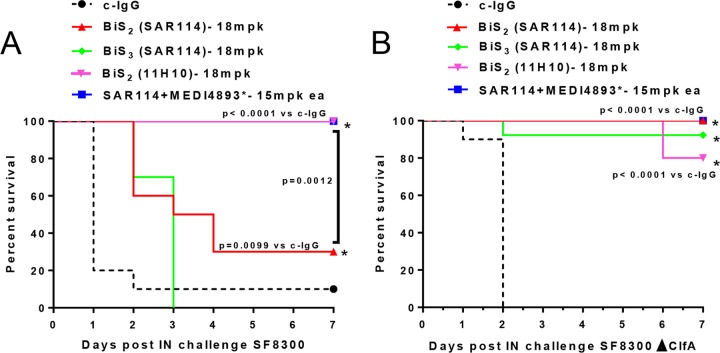
ClfA sequesters SAR114/MEDI4893* BiSAb in i.n. lethal pneumonia. C57/B6 mice (*n* = 10) were passively immunized i.p. with BiS_2_, BiS_3_, MEDI4893*, or the SAR114-MEDI4893* MAb combination at the indicated concentrations and i.n. infected 24 h later with 1.5e6 CFU of SF8300 (A) or the SF8300 Δ*clfA* isogenic mutant (B). Survival was monitored for 6 days. Results were analyzed with a log rank (Mantel-Cox) test. Results compared to those for c-IgG were considered statistically different at a *P* value of <0.05. Data are representative of three independent experiments.

Since SAR114, in MAb or either BiS format, binds ClfA001 with ∼1,000-fold greater affinity than 11H10 (Tables 1 and 3), we hypothesized that its binding to ClfA sequesters the BiSAb on the bacterial surface, leading to poorer capture and neutralization of alpha-toxin as it is secreted. Alpha-toxin is a key virulence factor in S. aureus pneumonia (20), and passive immunization with an anti-alpha-toxin MAb alone protects mice from lethal S. aureus pneumonia (6, 8, 21). Moreover, we previously showed that the anti-ClfA MAb does not impact survival in the pneumonia model, and anti-alpha-toxin–anti-ClfA provides protection similar to that of anti-alpha-toxin alone (9). Therefore, to determine if the decreased protection observed with the SAR114-BiS_2_Abs in the lethal bacteremia model resulted from inadequate alpha-toxin neutralization, we examined SAR114-BiS efficacy in the lethal pneumonia model. Passive immunization with MEDI4893* (15 mg/kg) alone or in combination with SAR114 resulted in 100% protection following challenge with SF8300. However, passive immunization with SAR114-BiS_2_ or -BiS_3_ resulted in 30 and 0% survival, respectively (Fig. 6A). Interestingly, passive immunization with the 11H10-BiS_2_, which has ∼1,000-fold reduced affinity for ClfA (Table 1), provided 100% survival. These results support the conclusion that binding to ClfA on the bacterial surface sequesters SAR114-BiSAbs, thus impairing alpha-toxin neutralization. To further test this hypothesis, mice were passively immunized with the BiS_2_ molecules prior to intranasal (i.n.) infection with the SF8300Δ*clfa* ClfA isogenic mutant. Prophylaxis with SAR114-BiSAb provided protection against the SF8300Δ*clfa* mutant similar to that of MEDI4893* (Fig. 6B). These results provide further evidence that SAR114-BiSAb binding to surface-localized ClfA prevents effective neutralization of soluble alpha-toxin.

**TABLE 3:** SAR114- and 11H10-BiS molecule affinity to ClfA variants

MAb and ClfA variant	Affinity to ClfA^*a*^		
	*K*_on_ (M^−1^s^−1^)	*K*_off_ (s^−1^)	*K_d_*
SAR114-BiS_2_			
ClfA001	1.04E+06	5.48E−06	5.283 pM
ClfA002	2.66E+06	9.69E−05	36.38 pM
ClfA004	3.16E+06	5.74E−06	1.815 pM
11H10-BiS_2_			
ClfA001	1.79E+06	5.00E−02	424.3 nM
ClfA002			ND
ClfA004	9.68E+04	4.00E−02	416.5 nM
SAR114-BiS_3_			
ClfA001	1.64E+06	1.56E−05	9.5 pM
ClfA002	1.85E+06	1.13E−04	61.1 pM
ClfA004	6.58E+05	3.40E−05	51.6 pM
11H10-BiS_3_			
ClfA001	4.60E+05	2.37E−02	51.4 nM
ClfA002	4.35E+03	1.26E−03	289.9 nM
ClfA004	1.39E+05	5.63E−03	40.3 nM

a The binding affinity constant (*K_d_*) of BiS molecules to the three ClfA variants was measured by Biacore as the ratio between dissociation (*K*_off_) and association (*K*_on_) kinetic rate constants. ND, not determined.

## DISCUSSION

S. aureus possesses a large arsenal of secreted and surface-expressed virulence factors, enabling it to cause a wide array of diseases, ranging from mild skin and soft-tissue infections to more severe and invasive diseases, such as pneumonia, osteomyelitis, and bloodstream infections (3). Differential expression of these virulence factors among clinical isolates and in different diseases presents a challenge when designing immunotherapeutic methods of treatment or prophylaxis and may be responsible in part for past clinical failures targeting individual S. aureus virulence determinants (22, 23). Therefore, immunotherapeutic strategies targeting multiple virulence pathways may block multiple pathogenic mechanisms and improve disease coverage, increasing the likelihood for clinical success (1, 24, 25).

Although alpha-toxin is a key virulence determinant in S. aureus pneumonia and skin and soft-tissue infections (20, 26), bloodstream infections are likely more complex, with a more complicated interplay of virulence determinants involved (27–31). Consistent with this hypothesis, prophylaxis with an anti-alpha-toxin IgG alone significantly reduced disease severity in S. aureus pneumonia and skin infection models in mice and rabbits (6, 8, 20, 21, 26, 32–34) but did not protect against challenge by all isolates tested in a lethal bacteremia model (Table 2) (9). However, passive immunization with MEDI4893* combined with anti-ClfA MAb 11H10 or SAR114 provided improved strain coverage, indicating that targeting multiple virulence factors may be necessary to protect against systemic S. aureus diseases such as bacteremia (Table 2; Fig. S3) (9).

ClfA is a fibrinogen-binding cell surface adhesin (35) that mediates bacterial agglutination and attachment to fibrinogen-coated surfaces and promotes complement evasion (36–40). Despite promising preclinical animal results with a limited number of strains (41, 42), an anti-ClfA MAb (tefibazumab [Aurexis]) and intravenous immunoglobulins (IVIG) enriched for high anti-ClfA titers (Veronate) failed to demonstrate a protective effect in clinical testing (43, 44). This may have resulted from targeting a single virulence factor, differential expression among clinical isolates, or ClfA sequence variation (16, 45). Indeed, Brady et al. recently reported on ClfA sequence-dependent epitopes that generated antibody responses following vaccination with reduced affinity to ClfA001 versus ClfA002 (46). When the anti-ClfA MAb 11H10, generated against ClfA001, was tested for binding to the 3 founder ClfA sequences (ClfA001, ClfA002, and ClfA004), it bound similarly to ClfA001 and ClfA004 but exhibited an ∼1,000-fold drop in affinity for ClfA002. This low affinity for ClfA002 resulted in a loss in functional activity (Fig. 1A and Table 1). However, this finding did not translate into a detectable loss of *in vivo* protection against the ClfA002-expressing ST5 strains tested (9). One possible explanation for this result is high-avidity binding on the bacterial surface compensating for a loss in MAb affinity for ClfA. Nevertheless, since ClfA002-expressing ST5/USA100 strains are frequently associated with bloodstream infections (47, 48), a campaign was initiated to identify a more broadly reactive high-affinity anti-ClfA MAb from human tonsillar memory B cells (49). The lead MAb identified from this campaign, SAR114, exhibited at least a 100-fold greater affinity for the 3 founder ClfA genotypes than 11H10 (Table 1), improved *in vitro* functional activity against ClfA002 (Fig. 1B)- and ClfA002-expressing strains (Fig. 1C), and broad strain coverage in lethal bacteremia when combined with an anti-alpha-toxin MAb, MEDI4893* (Table 2; Fig. S3).

The benefits of MAb or vaccine antigen combinations over targeting individual antigens have also been reported for other pathogens. In fact, it was shown that combining MAbs with multiple mechanisms of action on a single BiSAb can provide improved protective activity not only over the individual MAbs but also the MAb combination (10, 50–52). In addition to the potential for improved efficacy of a BiSAb, a single-molecule approach could simplify Ab manufacturing (e.g., expression and purification) and overcome regulatory hurdles associated with clinical development of MAb combinations (10, 53). Here, anti-alpha-toxin–anti-ClfA BiSAbs (except SAR114-BiS_3_, which lost some alpha-toxin-neutralizing activity) exhibited activity *in vitro* similar to that of the parental IgG1 MAbs (Fig. 4A and B); however, they provided no benefit over the MAb combinations *in vivo*, and the BiS molecules constructed with the higher-affinity anti-ClfA MAb (SAR114) even lost protective activity relative to the MAb combination (Fig. 5A and B). This did not result from alpha-toxin or ClfA simply competing for BiSAb binding, because the BiSAbs retained full activity *in vitro* in the presence of a 10-molar excess of either ClfA or alpha-toxin (Fig. 4C and D). The *in vivo* activity loss likely resulted from SAR114 high-affinity binding to ClfA sequestering the BiSAb on the bacterial surface, where it is apparently less effective at capturing and neutralizing alpha-toxin as it is secreted. This conclusion is supported by the finding that SAR114-BiS_2_Ab retained full alpha-toxin neutralization activity in mice infected with SF8300 lacking ClfA (Δ*clfA*) (Fig. 6B).

In this work, we also compared the activity of two BiS constructs with different antibody pairs targeting the same antigens. The BiS constructs differed in that the anti-alpha-toxin scFv was fused to either the N (BiS_2_) or C terminus (BiS_3_) of the heavy chain. Both 11H10-BiS_2_ and -BiS_3_ molecules retained full anti-ClfA and anti-alpha-toxin neutralizing activity. Alternatively, SAR114-BiS_3_ lost alpha-toxin-neutralizing activity, indicating that the best-performing BiS molecule in some cases is independent of antigen-specific scFv location. These results highlight the fact that IgG characteristics, such as antigen specificity, affinity, location, and distance between Fab and scFv, may not be used to predict BiSAb activity, but the best BiS format must be selected empirically for the individual IgG pairs. MEDI3902 is an example of an antibacterial BiSAb against P. aeruginosa that binds the abundant cell surface polysaccharides Psl and PcrV of the type 3 secretion system (T3SS). Like the anti-alpha-toxin–anti-ClfA combination, MEDI3902 exhibits multiple mechanisms of action, including opsonophagocytic killing, anti-adherence activity (Psl), and inhibition of T3SS-mediated cytotoxicity. In contrast to the anti-ClfA–anti-alpha-toxin BiSAbs, MEDI3902 exhibited improved T3SS neutralization activity *in vitro* and increased protection in a murine pneumonia model relative to the MAb combination (10). The increased potency of MEDI3902 was hypothesized to result from its low-affinity binding to the highly abundant surface polysaccharide Psl, thereby increasing the local concentration and high-affinity binding and subsequent neutralization of the low-abundance surface target, PcrV (10). Such binding to two distinct surface antigens with differential affinities likely accounts for the improved activity seen with MEDI3902. *In vitro* and *in vivo* assays comparing efficacies between different BiS molecules, where anti-PcrV MAb is in Fab and anti-Psl is an scFv linked in N-terminal (BiS_2_), C-terminal (BiS_3_), and upper hinge (BiS_4_) region, showed better activity for BiS_4_Ab than the other constructs. This suggested that interparatopic distance is important for MEDI3902 activity (10). In contrast, the SAR114-BiSAbs, where MEDI4893* is grafted either to the N terminus (BiS_2_) or C terminus (BiS_3_) of the molecule, bind with higher affinity to surface-localized ClfA, which likely sequesters the BiSAb to the bacterial surface and prevents it from optimally neutralizing alpha-toxin.

Taken together, these results support the rationale for the use of a multimechanistic MAb strategy to provide the most potent and broadly protective activity against S. aureus bacteremia. However, not only does the combination of anti-ClfA and anti-alpha-toxin binding specificities on one BiSAb demonstrate no benefit over the MAb mixture, but also, when a high-affinity anti-ClfA MAb was used, the BiS showed reduced *in vivo* activity. This finding illustrates that a bispecific approach to a MAb combination cannot be universally applied to all antibacterial MAb combinations. The decision to use a MAb combination or a BiSAb is likely dependent on many factors, including antigen location (surface-expressed or secreted), antigen concentration or density, and relative MAb component binding affinities, along with developmental and regulatory concerns. This highlights the need to empirically determine the MAb format independently for each desired application.

## MATERIALS AND METHODS

### Bacterial strains.

Community-acquired MRSA SF8300 (USA300) and its isogenic ClfA deletion mutant (Δ*clfa*) were previously described (23). NRS strains were obtained from the Network on Antimicrobial Resistance in Staphylococcus aureus (NARSA). ARC strains were obtained from the Astra Zeneca Research Collection, and strains identified with a number beginning with 304 were obtained from Eurofins. All strains and their corresponding multilocus sequence types (MLST) and ClfA genotypes are summarized in Table S1 in the supplemental material.

### Whole-genome sequencing and analysis.

DNA was purified from bacterial cultures via bead beating followed by extraction using a PureLink genomic DNA minikit (ThermoFisher). Sequencing libraries were prepared by Covaris mechanical shearing followed by a NEBNext Ultra DNA library preparation kit for Illumina (New England BioLabs Inc.). Sequencing was performed via MiSeq 2 × 250 runs (Illumina) with a targeted depth of 150-fold. Reads were *de novo* assembled (CLCBio Genomic Workbench 9.0.1), and *clfA* open reading frames were extracted and translated to protein sequences. ClfA protein alleles in the present study were matched to the allele numbering by Murphy et al. (16) by comparing all sequences (this study and GenBank accession numbers HQ424254 to HQ424312) to a common reference, USA300_FPR3757. MLSTs were determined using SRST2 (54) and the S. aureus MLST profiles (http://pubmlst.org).

### ClfA MAb generation.

Memory B cells were isolated from cryopreserved lymphocytes isolated from tonsils using phycoerythrin (PE)-Cy7-labeled CD19 microbeads (BD Biosciences), followed by staining with anti-PE beads (Miltenyi Biotec) and depletion of cells carrying IgM, IgD, and IgA by cell sorting on a FACSAria (BD Biosciences). Cells were immortalized under clonal conditions with Epstein-Barr virus as described previously (55). After 2 weeks, the culture supernatants were screened for the presence of ClfA001-specific MAbs using a 384-well-based ELISA. Positive cultures were expanded in complete RPMI medium and selected for their ability to bind to ClfA genotypes 001, 002, and 004 with high affinity. The VH and VL sequences were retrieved by reverse transcription-PCR (RT-PCR).

### Cloning and expression of the ClfA variants.

ClfA sequences corresponding to the N2N3 domain (amino acids 221 to 559) of ClfA genotypes 001, 002, and 004 were obtained from GenBank (accession numbers HG424254, HQ424255, and HQ424257) (16). DNA encoding each sequence was codon optimized and subcloned into pET28a(+) with an AviTag biotinylation signal (GLNDIFEAQKIEWHE) cloned into the 5′ end of the *clfA* coding sequence and expressed in Escherichia coli BL21(DE3). Biotinylation was induced after cotransformation of BL21(D3) cells with an isopropyl-β-d-thiogalactopyranoside (IPTG)-inducible plasmid containing the *birA* gene, encoding biotin ligase, and AviTag-ClfA containing pET28a(+). Recombinant proteins were purified from E. coli lysates using a HisTrap HP column (GE Healthcare) equilibrated with buffer A (25 mM sodium phosphate, pH 7.8, with 500 mM NaCl). The column was washed with buffer A followed by 1% 3-[(3-cholamidopropyl)-dimethylammonio]-1-propanesulfonate in buffer A for endotoxin removal. Impurities were removed with a 25 mM imidazole (Teknova) wash, and then ClfA was eluted with a linear gradient to 500 mM imidazole. The ClfA-containing fractions were pooled and dialyzed into Q buffer A (25 mM Tris, pH 7.5). The lysates were then loaded onto a 5-ml HiTrap Q Sepharose HP column (GE Healthcare) and eluted with a linear gradient to 500 mM NaCl. Fractions containing pure ClfA were dialyzed into 1× phosphate-buffered saline (PBS), pH 7.2, and aliquots were frozen at −80°C.

### Bispecific antibodies.

Bispecific antibodies were generated as previously described (11, 14). Briefly, anti-ClfA MAb 11H10 or SAR114 was used as the IgG scaffold, and MEDI4893* was grafted in scFv format. MEDI4893* scFv was synthesized in the VL-VH format with a 20-amino-acid (GGGGSx4) linker between the light and heavy variable domains (GeneArt, Life Technologies, IL). BiS_2_Abs were constructed by fusing MEDI893* scFv sequences to the N terminus of the heavy chain of 11H10 or SAR114 anti-ClfA IgG1. BiS_3_ constructs were generated by appending the linker-scFv of MEDI4893* to the C terminus of the heavy chain of 11H10 or SAR114. BiS_2_ and BiS_3_ molecules were expressed by transient transfection in 293 cells, purified by protein A affinity chromatography, and polished by size exclusion chromatography. The integrity of each molecule was assessed by mass spectrophotometry and by intact mass and peptide mapping to verify proper formation of engineered and endogenous disulfide bounds.

### Hemolytic assay.

Rabbit red blood cell (RBC) hemolytic assay was performed as described previously (56). Briefly, serial dilutions of the BiSAbs and MEDI4893* (500 to 1.7 nM) were mixed with alpha-toxin (0.1 μg/ml = 3 nM) and incubated with 50 μl of washed rabbit RBC (Peel Freeze) for 1 h at 37°C. In some assays, anti-alpha-toxin scFv of BiSAb was saturated with a 10 M excess of ClfA (5 μM). Plates then were centrifuged at 1,200 rpm for 3 min, and 50 μl of supernatant was transferred to new plates. Nonspecific human IgG1 R347 was used as a negative control (c-IgG) (56). The optical density at 450 nm (OD_450_) was measured with a spectrophotometer (Molecular Devices). Inhibition of hemolysis was calculated as 100 − [100 × (OD_alpha-toxin+MAb_/OD_alpha-toxin_)].

### Fibrinogen binding assay.

Nunc MaxiSorp plates (Thermo Fisher Scientific) were coated overnight at 4°C with 2 μg/ml human fibrinogen (Sigma), washed 3× with PBS containing 0.1% Tween 20 (wash buffer), and blocked for 1 h at room temperature (RT) with 200 μl/well casein (Thermo Fisher). Following 3 washes, the plates were incubated for 1 h at room temperature with a mix of 50 μl AviTag ClfA_221–559_ (2 μg/ml) and serial dilutions of anti-ClfA MAb or BiSAb in a 100-μl final volume of PBS. In some assays, anti-ClfA IgG1 of BisAb was saturated with a 10 M excess of alpha-toxin (6.6 mM). After washes, bound ClfA was detected using horseradish peroxidase (HRP)-conjugated streptavidin (1:20,000; GE Healthcare) and then 100 μl 3,3′,5,5′-tetramethylbenzidine (TMB) substrate (KPL). The reaction was stopped after 10 min with 100 μl 0.2 M H_2_SO_4_. The OD_450_s on plates were read on a spectrophotometer.

The percent inhibition of ClfA binding to fibrinogen was calculated with the following formula: 100 − [100 × (OD_ClfA+MAb_/OD_ClfA,no MAb_)].

### Agglutination inhibition in human plasma.

S. aureus clinical isolates were cultured overnight in Tryptic soy broth, washed in PBS, and suspended to one-tenth of the original volume in ice-cold PBS. Anti-ClfA MAbs or BiSAbs were serially diluted (2-fold) in 30 μl PBS starting at 200 μg/ml and mixed with 30 μl of citrated human plasma in a 96-well U-bottom plate (Thermo Fisher Scientific). Bacteria were added (30 μl) and incubated for 5 min at 37°C. Each well was evaluated visually, and the lowest MAb concentration where bacteria agglutinated was recorded. R347, a human anti-gp120 MAb, was utilized as an isotype control human IgG1 (c-IgG).

### Opsonophagocytic killing assay.

HL-60 cells were obtained from ATCC (Manassas, VA), and OPK assay was conducted as previously described (9).

### Measurement of kinetic rate and binding constants by Biacore.

Kinetic rate constants (*K*_on_ and *K*_off_) for binding of the anti-ClfA MAbs to ClfA genotypes ClfA001, ClfA002, and ClfA004 were measured using an IgG capture assay CM4 sensor chip with a final surface density of 500 resonance units (RUs) on a Biacore T200 instrument (GE Healthcare Life Sciences). A reference flow cell surface was also prepared on a CM4 sensor chip using the identical immobilization protocol without MAbs. Anti-ClfA MAbs were prepared at 25 nM (100 nM for Bis_2_-SAR114) in immobilization buffer (10 mM sodium acetate, pH 4), along with 3-fold serial dilutions of ClfA (0.009 nM to 20 nM for SAR114 and Bis_2_-SAR114; 0.91 to 2,000 nM ClfA001 and 2.28 to 5,000 nM ClfA002 and ClfA004 for 11H10 and Bis_2_-11H10) in instrument buffer (HBS-EP buffer; 0.01 M HEPES, pH 7.4, 0.15 M NaCl, 3 mM EDTA, and 0.005% P-20). Each concentration of ClfA was first injected over the immobilized MAbs and reference surfaces at a flow rate of 75 μl/min for 60 s. The resulting binding response curves yielded the association phase data. Following the injection of ClfA, the flow was then switched back to instrument buffer for 15 min (3 h for 2 highest concentrations of ClfA001) to permit collection of dissociation phase data, followed by a 1-min pulse of 10 mM glycine, pH 1.7, to regenerate the IgG-immobilized surface on the chip. Binding responses from duplicate injections of each concentration of ClfA were recorded for all MAbs. In addition, several buffer injections were interspersed throughout the injection series. Select buffer injections were used along with the reference cell responses to correct the raw data sets for injection artifacts and/or nonspecific binding interactions. Fully corrected binding data were then fit to a 1:1 binding model (Biacore T200 evaluation software, version 2.0; Biacore, Inc.) that included a term to correct for mass transport-limited binding, should it be detected. These analyses determined the kinetic rate constants *K*_on_ and *K*_off_, from which the apparent dissociation constant (*K_D_*) was calculated as *K*_off_/*K*_on_.

### Competition binding.

Anti-ClfA MAbs 11H10 and SAR114 were tested in a competition assay for binding to ClfA001 on an Octet RED96 system (FortéBio). Briefly, MAbs diluted at 5 μg/ml in PBS were captured on an aminopropylsilane (APS) biosensor for 7 min. Coated biosensors were moved into blocking buffer-containing wells (PBS, 1 mg/ml bovine serum albumin [Sigma]) for 6 min to block free sensor binding sites, subsequently incubated for 7 min with 2.5 μg/ml ClfA001 diluted in blocking buffer, and finally moved into wells containing the competing MAbs diluted at 5 μg/ml in blocking buffer. Data were analyzed using the Octet data acquisition and analysis software. The absence of association of the competing MAb resulted in competition and, thus, recognition of the same antigenic site, while noncompetition is obtained when association of the second MAb was detected.

### Mouse models.

All experiments were performed in accordance with institutional guidelines following experimental protocol review and approval by the Institutional Biosafety Committee (IBC) and the Institutional Animal Care and Use Committee (IACUC) at MedImmune.

### Lethal bacteremia.

Groups of 10 6- to 8-week-old female BALB/c mice (Harlan) were passively immunized by intraperitoneal (i.p.) injection of c-IgG, BiS molecules, or the anti-ClfA MAb–MEDI4893* combination and then challenged 24 h later by intravenous (i.v.) injection with the 90% lethal dose (LD_90_) of each S. aureus isolate. Survival was monitored for 2 weeks. Statistical analysis of a specific antistaphylococcal antigen versus c-IgG was performed with a log rank (Mantel-Cox) test. Data were considered statistically different at a *P* value of <0.05, indicated with an asterisk.

### Pneumonia.

Female C57/B6 mice (Jackson) were injected i.p. with MEDI4893* or BiS molecule, and pneumonia was induced by intranasal infection with SF8300 (1e8 CFU) as described previously (8). Animal survival was monitored for 6 days. Statistical analysis versus c-IgG was performed with a log rank (Mantel-Cox) test. Data were considered statistically different at a *P* value of <0.05, indicated with an asterisk.

## Supplementary Material

Supplemental material
