# Supplementary material for: Multimechanistic Monoclonal Antibodies (MAbs) Targeting Staphylococcus aureus Alpha-Toxin and Clumping Factor A: Activity and Efficacy Comparisons of a MAb Combination and an Engineered Bispecific Antibody Approach
Source: Antimicrob Agents Chemother. 2017 Jul 25;61(8):e00629-17. doi: 10.1128/AAC.00629-17 (PMC5527613; doi:10.1128/AAC.00629-17)
Supplement: Supplemental material [file supp_61_8_e00629-17__index.html]

Supplemental material 

# Multimechanistic Monoclonal Antibodies (MAbs) Targeting Staphylococcus aureus Alpha-Toxin and Clumping Factor A: Activity and Efficacy Comparisons of a MAb Combination and an Engineered Bispecific Antibody Approach

## Supplemental material

- Supplemental file 1 -

  Supplemental Tables S1 and S2 and Figures S1 to S5

  PDF, 383K
