# Supplementary material for: Multimechanistic Monoclonal Antibodies (MAbs) Targeting Staphylococcus aureus Alpha-Toxin and Clumping Factor A: Activity and Efficacy Comparisons of a MAb Combination and an Engineered Bispecific Antibody Approach
Source: Antimicrob Agents Chemother. 2017 Jul 25;61(8):e00629-17. doi: 10.1128/AAC.00629-17 (PMC5527613; doi:10.1128/AAC.00629-17)
Supplement: Supplemental material [file AAC.00629-17_zac008176417s1.pdf]

Table S1 SAR114 coverage against 112 isolates representing 40 different sequence type (ST) in human plasma agglutination assay.

Strain	ST	ClfA variant	SAR114 (μg/ml)	Strain	ST	ClfA variant	SAR114 (μg/ml)
3048965	1	ClfA044	1.56	3048984	12	ClfA004	0.78
3049036	1	ClfA012	6.2	3048948	12	ClfA043	3.1
3049040	1	ClfA012	3.1	3049084	12	ClfA017	1.56
3049054	1	ClfA046	6.2	3049092	15	ClfA015	1.56
3049086	1	ClfA055	1.56	3049113	15	ClfA015	1.56
3049101	1	ClfA055	3.1	3049120	15	ClfA015	0.78
NRS123	1	ClfA012	12.5	3049172	15	ClfA015	0.78
ARC801	NA	NA	3	3049178	15	ClfA015	1.5
ARC4211	5	ClfA002	1.5	3048963	15	ClfA015	1.56
NRS387	5	ClfA002	1.5	3049077	15	ClfA015	3.1
ARC2378	5	ClfA002	3.12	3049090	22	ClfA022	1.56
ARC2379	5	ClfA002	3.1	3049125	22	ClfA053	1.56
ARC2380	5	ClfA002	1.56	3049126	22	ClfA053	1.56
ARC634	5	ClfA002	1.56	3049127	22	ClfA022	3.1
ARC635	5	ClfA002	0.7	3049129	22	ClfA022	1.56
ARC795	5	ClfA002	6.25	3049046	26	ClfA005	0.78
NRS382	5	ClfA002	3	3049089	30	ClfA018	0.78
3049005	5	ClfA002	3.1	3048989	30	ClfA004	1.56
3049139	7	ClfA018	1.56	3049088	30	ClfA004	0.78
3049166	7	ClfA018	3.1	3049093	30	ClfA004	1.56
ARC796	8	ClfA001	1.5	3049096	30	ClfA004	0.78
3043406	NF	ClfA001	25	3049114	30	ClfA004	1.56
3043527	8	ClfA001	6	3049173	30	ClfA004	0.78
3043691	NA	ClfA001	6.25	3049174	30	ClfA004	1.56
3049057	8	ClfA001	6	3049176	30	ClfA004	1.56
ARC2068	8	ClfA001	3.12	3049191	30	ClfA047	1.56
ARC2402	8	ClfA001	3.12	ARC2081	30	ClfA004	0.7
ARC2403	8	ClfA001	6.25	ARC629	30	ClfA004	1.56
ARC2464	8	ClfA001	3.12	UAMS-1	30	ClfA004	1.56
ARC2465	8	ClfA001	3.12	3049095	34	ClfA004	0.78
ARC517	8	ClfA001	3.12	3049118	45	ClfA009	0.78
BAA-1556	8	ClfA001	6	3049119	45	ClfA009	0.78
SF8300	8	ClfA001	1.5	3049122	45	ClfA009	1.56
NRS384	8	ClfA001	1.56	3049135	45	ClfA056	1.56
ARC2490	8	ClfA001	3.12	3049141	45	ClfA009	12.5
ARC2491	8	ClfA001	3.12	NRS22	45	ClfA009	1.5
ARC2558	8	ClfA001	3.12	3049048	45	ClfA009	3
NRS385	8	ClfA001	6.25	3049112	45	ClfA009	1.5
Newman	8	ClfA001	1.5	3049064	45	ClfA009	0.78

**Strain**	**ST**	**ClfA variant**	**SAR114 (μg/ml)**
3049048	45	NT	1.56
3048969	59	ClfA014	1.56
ARC2566	72	ClfA049	3.12
3049091	72	ClfA016	1.56
3049042	87	ClfA014	3.1
ARC2408	87	ClfA014	0.78
3049032	88	ClfA007	3.1
3049167	96	ClfA045	0.78
3048966	97	ClfA001	1.56
3049033	97	ClfA001	0.78
3049137	97	ClfA001	1.56
3049134	101	ClfA029	0.78
3049138	106	ClfA029	3.1
3049103	121	ClfA008	0.78
3049181	121	ClfA052	1.56
ARC2784	188	ClfA019	3
3049179	188	ClfA019	3.1
3049047	188	ClfA019	6.2
3049162	188	ClfA058	5
ARC797	225	ClfA002	6
3049152	239	ClfA003	3.1
NRS383	346	ClfA004	6
3048952	398	ClfA048	0.78
3049196	764	ClfA002	0.78
3049177	769	ClfA012	0.78
ARC516	890	ClfA054	1.56
3049104	1210	ClfA042	1.56
Wright	508	ClfA009	3.1
Lowenstein	25	ClfA005	3.1
3049109	582	ClfA013	3.1
3049115	NF	ClfA015	0.78
3049182	NF	ClfA050	1.56
3049163	NF	ClfA058	5
3049168	NF	ClfA058	12.5

Each isolate was grown overnight, and incubated for 3 min at 37°C with serial dilution of anti-ClfA mAb SAR114 (50 to 0.7 μg/ml), and human sera. Minimum concentration of SAR114 (μg/ml) resulting in agglutination inhibition was recorded. For each isolate, no inhibition for c-IgG>100 μg/ml. In some case, strains were non typeable (NT) as indicated. Whole genome sequence was not performed on some strains, therefore ST and ClfA variant sequence were unknown (NA).

Table S2 In vitro activities as measured in IC_{50} (μM) of SAR114- and 11H10-Bis molecules in fibrinogen binding inhibition and rabbit RBC hemolytic assays

	Fibrinogen binding[a]						RBC hemolysis[b]	
				+AT (6.6mM)				+ClfA001 (1mM)
	ClfA001	ClfA002	ClfA004	ClfA001	ClfA002	ClfA004	AT	AT
**11H10**	24.59	997.5	23.94	18.34	1239	32.7		
**11H10-BiS2**	29.01	1515	22.26	36.25	1324	32.15	0.138	0.146
**11H10-BiS3**	24.86	761.2	18.65	44.18	979.4	37.5	0.138	0.149
**SAR114**	16.09	6.004	13.61	12.49	6.37	20.64		
**SAR114-BiS2**	25.7	38.7	21.86	28.37	25.64	19.61	0.09	0.157
**SAR114-BiS3**	36.35	23.4	29.85	26.96	22.8	28.11	1.052	1.126
**MEDI4893***							0.1	0.02

Comparison of vitro activities of 11H10- or SAR114-BiS molecules with parental mAbs 11H10 and SAR144 was measured in the fibrinogen binding assay[a] to the three main ClfA genotypes alone (666μM) or in 10M excess of AT (6.6 mM), and with MEDI4893* in the rabbit RBC hemolytic assay[b] with AT alone (0.1 μM) or in presence of 10M excess of ClfA001 (1 μM). IC50 (μM) was calculated as the Ab concentration resulting in 50% inhibition in each assay.

Fig. S1

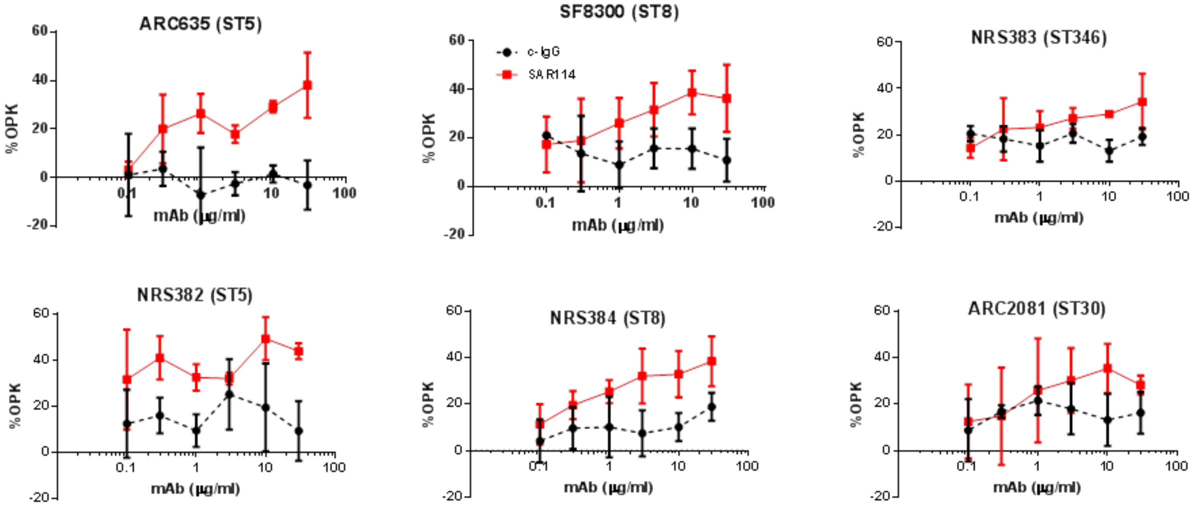

Fig.S1: Opsonophagocytic killing activity of anti-ClfA mAb SAR114 against different isolates. *S. aureus* strains were incubated with human HL-60 cells, human sera, and serial dilutions of SAR114 (■) or c-IgG (●). Graph represents mean values ± SD of three independent experiments.

Fig. S2

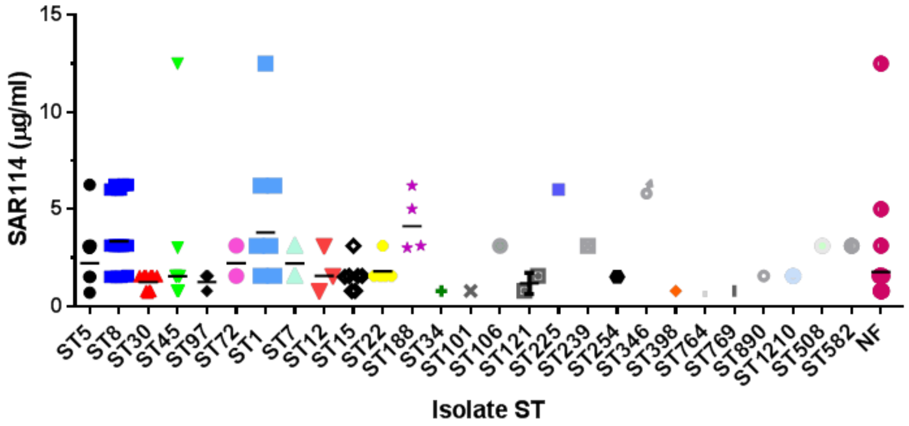

Figure S2: Anti-ClfA mAb SAR114 inhibits agglutination of multiple sequence type strains. One hundred and twelve *S. aureus* clinical isolates representing 40 different sequence types (ST) and some not found (NF) were cultured overnight in TSB, washed in PBS and suspended to one tenth of the original volume in ice-cold PBS. Anti-ClfA mAb SAR114 was two-fold serially diluted starting at 200µg/ml and mixed with 30µl of citrated human plasma in a 96-well U bottom plate (Thermo Fisher Scientific). Bacteria were added (30µl) and incubated 5min at 37°C. Each well was evaluated visually and the lowest mAb concentration where bacteria agglutinated was recorded. R347, a human anti-gp120 mAb was utilized as an isotype control human IgG1 (c-IgG).

Fig. S3

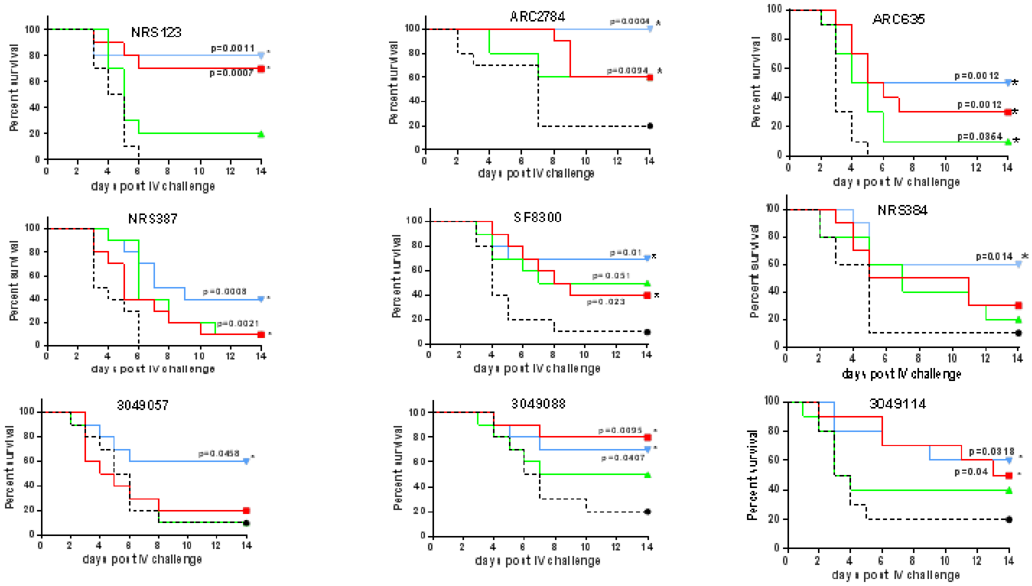

Fig. S3: SAR114+MEDI4893* provides strain coverage in IV lethal bacteremia. Balb/c mice were immunized with 15mpk of SAR114 (■), MEDI4893* (▲) or a combination of each at 7.5mpk (▼), and IV infected 24h later with different *S. aureus* clinical isolates from diverse sequence types (ST), and ClfA genotypes: NRS123 (ST1, ClfA012), NRS387 (ST5, ClfA002), ARC635 (ST5, ClfA002), 3049043 (ST5, ClfA002), 3049057 (ST8, ClfA001), SF8300 (ST8, Clf A001), 3049088 (ST30, ClfA004), 3049114 (ST30, ClfA004) and ARC2784 (ST188, ClfA019). Irrelevant c-IgG (●) was injected at 15mpk. Survival was monitored for 2 weeks. Results were analyzed with a Log Rank (Mantel Cox) test. Statistical analysis versus c-IgG were considered statistically different if $p<0.05$, and indicated with an asterix (*). Data are representative of three independent experiments.

Fig. S4

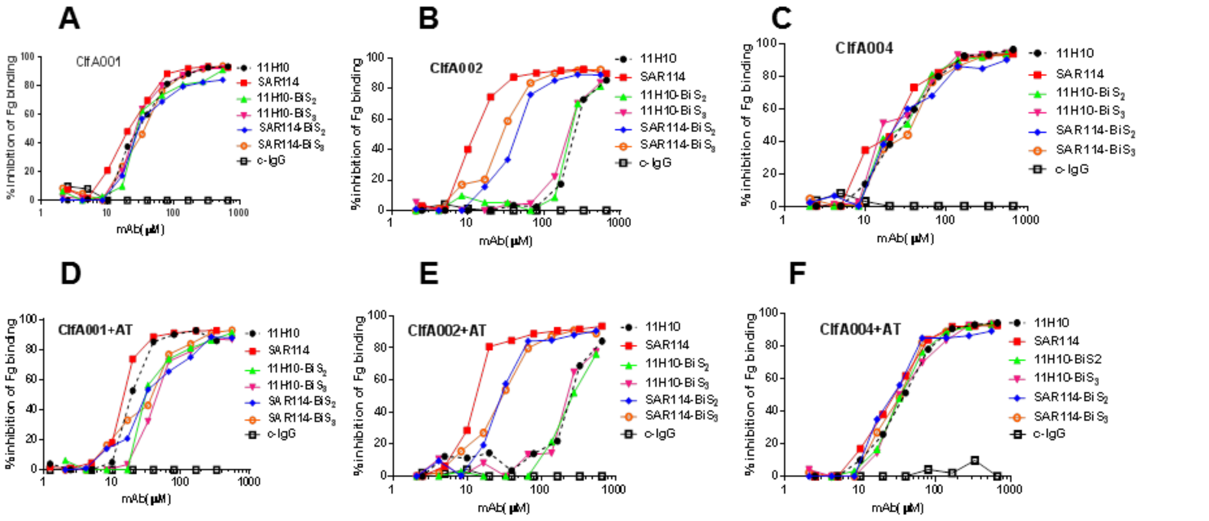

Fig. S4: ClfA mAb and BiSAb activity in fibrinogen inhibition assay. Inhibition of fibrinogen binding to the three main ClfA genotypes was measured in presence of serial dilutions of 11H10, SAR114 or respective BisAbs (A, B, C). Similar assay conducted when AT scFv was saturated in presence of 10M excess of AT (6.6mM) (D, E, F). Graphs are representative of one of three separate experiments.

Fig. S5

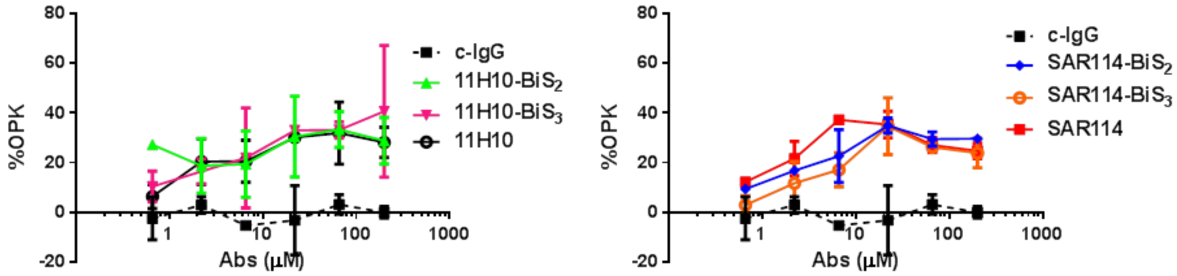

Figure S5: Opsonophagocytic killing (OPK) activity of anti-ClfA/AT BiS molecules. *S. aureus* Newman isolate was incubated with human HL-60 cells, human sera, and serial dilutions of SAR114 or 11H10-BiS molecules, SAR114 and 11H10 parental mAbs or negative control-IgG (■). Graph represents mean values ± SD of two independent experiments.
